# Supplementary material for: Dynamics of T-cell Responses Following COVID-19 mRNA Vaccination and Breakthrough Infection in Older Adults
Source: Pathog Immun. 2023 Nov 17;8(1):117–35. doi: 10.20411/pai.v8i1.613 (PMC10686373; doi:10.20411/pai.v8i1.613)
Supplement: Supplementary Tables 1–5 [file pai-8-117-s01.pdf]

PATHOGENS AND IMMUNITY
IN ASSOCIATION WITH CASE WESTERN RESERVE UNIVERSITY AND THE RICHARD J. FASENMYER FOUNDATION

SUPPLEMENTARY TABLES

Supplementary Table 1: Multivariable Zero-inflated Beta Regressions Exploring the Relationship Between Sociodemographic, Health and Vaccine-related Variables on Spike-specific T-cell Responses Following 2 COVID-19 mRNA Vaccine Doses

Variable	Outcome measure					
	% Spike-responsive CD4+ T-cells after 2 vaccine doses			% Spike-responsive CD8+ T-cells after 2 vaccine doses		
	**Estimate**	**Standard error**	***P*-value**	**Estimate**	**Standard error**	***P*-value**
Age (per year)	0.0043	0.0043	0.32	0.0014	0.0046	0.75
Male sex	0.12	0.17	0.46	0.038	0.18	0.83
White ethnicity	-0.38	0.17	**0.03**	0.039	0.18	0.83
Major health conditions (versus none)[a]	-0.22	0.31	0.49	-0.18	0.34	0.59
Other health conditions (versus none)[b]	-0.009	0.18	0.96	-0.084	0.19	0.67
mRNA-1273-containing initial vaccine regimen[b]	-0.064	0.25	0.80	-0.013	0.26	0.96
Days between 1st and 2nd vaccine dose	0.0033	0.0033	0.33	0.0040	0.0037	0.28

[a]Binary variable, defined as one or more of: chronic blood disorder or cancer, versus no such condition.
[b]Binary variable, defined as one or more of: hypertension, diabetes, asthma, obesity (body mass index ≥30), chronic diseases of lung, liver, kidney or heart, versus no such conditions.
[c]Defined as two doses of mRNA-1273, or a heterologous regimen consisting of one dose each of mRNA-1273 and BNT162b2 (vs a reference group defined as two doses of BNT162b2).

Datwani S, Kalikawe R, Mwimanzi K, Speckmaier S, Liang R, Sang Y, Waterworth R, Yaseen F, Lapointe HR, Barad E, DeMarco ML, Holmes DT, Simons J, Montaner JSG, Romney MG, Brumme ZL, Brockman MA. Dynamics of T-cell Responses Following COVID-19 mRNA Vaccination and Breakthrough Infection in Older Adults. *Pathogens and Immunity.* 2023;8(1):117–135. doi: 10.20411/pai.v8i1.613

PATHOGENS AND IMMUNITY
IN ASSOCIATION WITH CASE WESTERN RESERVE UNIVERSITY AND THE RICHARD J. FASENMYER FOUNDATION

Supplementary Table 2: Multivariable Zero-inflated Beta Regressions Exploring the Relationship Between Sociodemographic, Health and Vaccine-related Variables on Spike-specific T-cell Responses Following 3 COVID-19 mRNA Vaccine Doses

Variable	Outcome measure					
	% Spike-responsive CD4+ T-cells after 3 vaccine doses			% Spike-responsive CD8+ T-cells after 3 vaccine doses		
	**Estimate**	**Standard error**	***P*-value**	**Estimate**	**Standard error**	***P*-value**
Age (per year)	-0.0034	0.0034	0.32	0.0025	0.0043	0.56
Male sex	0.29	0.11	**0.007**	0.098	0.13	0.45
White ethnicity	-0.17	0.10	0.08	-0.11	0.12	0.35
Major health conditions (versus none)[a]	0.33	0.20	0.10	0.18	0.24	0.44
Other health conditions (versus none)[b]	0.23	0.11	**0.03**	-0.12	0.13	0.36
mRNA-1273-containing initial vaccine regimen[c]	0.061	0.15	0.69	0.08	0.19	0.67
Days between 1st and 2nd vaccine dose	0.0042	0.0031	0.18	0.003	0.004	0.45
mRNA-1273 as third vaccine dose[d]	0.24	0.11	**0.02**	0.073	0.12	0.54
Days between 2nd and 3rd vaccine dose	0.0025	0.0019	0.19	0.000053	0.0025	0.98
% Spike responsive T-cells after two doses (per 1% increment)[e]	0.71	0.066	**$2x10^{-16}$**	0.78	0.086	**$6x10^{-14}$**

[a]Binary variable, defined as one or more of: chronic blood disorder or cancer, versus no such condition.
[b]Binary variable, defined as one or more of: hypertension, diabetes, asthma, obesity (body mass index ≥30), chronic diseases of lung, liver, kidney or heart, versus no such conditions.
[c]Defined as two doses of mRNA-1273, or a heterologous regimen consisting of one dose each of mRNA-1273 and BNT162b2 (vs. a reference group defined as two doses of BNT162b2).
[d]Reference group defined as a BNT162b2 third vaccine dose.
[e]Post-second dose % CD4+ and CD8+ T-cell responsiveness values were used for the post-third dose CD4+ and CD8+ T-cell models, respectively.

Datwani S, Kalikawe R, Mwimanzi K, Speckmaier S, Liang R, Sang Y, Waterworth R, Yaseen F, Lapointe HR, Barad E, DeMarco ML, Holmes DT, Simons J, Montaner JSG, Romney MG, Brumme ZL, Brockman MA. Dynamics of T-cell Responses Following COVID-19 mRNA Vaccination and Breakthrough Infection in Older Adults. *Pathogens and Immunity.* 2023;8(1):117–135. doi: 10.20411/pai.v8i1.613

PATHOGENS AND IMMUNITY
IN ASSOCIATION WITH CASE WESTERN RESERVE UNIVERSITY AND THE RICHARD J. FASENMYER FOUNDATION

Supplementary Table 3. Associations Between HLA Class I Alleles and SARS-CoV-2 Spike-specific CD8+ T-cell Responses After 3 COVID-19 mRNA Vaccine Doses

Allele	N with[a]	N without	median with[b]	median without	*P*-value[c]	q-value
**B*39:01**	**5**	**85**	**0.27**	**0.77**	**0.004**	**0.08**
**A*02:03**	**5**	**85**	**2.50**	**0.73**	**0.01**	**0.08**
**B*44:03**	**9**	**81**	**0.46**	**0.78**	**0.01**	**0.08**
**C*07:01**	**17**	**73**	**0.57**	**0.78**	**0.02**	**0.13**
C*12:03	5	85	1.70	0.76	0.06	0.27
B*57:01	5	85	1.10	0.76	0.11	0.40
B*46:01	8	82	0.93	0.75	0.14	0.41
C*01:02	15	75	0.87	0.73	0.18	0.41
B*51:01	12	78	1.21	0.76	0.18	0.41
C*02:02	6	84	0.49	0.77	0.20	0.41
B*14:02	5	85	0.78	0.73	0.20	0.41
B*08:01	8	82	0.60	0.77	0.21	0.41
A*03:01	12	78	0.64	0.77	0.29	0.49
B*58:01	5	85	0.90	0.76	0.31	0.49
A*24:02	24	66	0.74	0.77	0.32	0.49
C*03:02	6	84	0.88	0.75	0.36	0.51
A*11:01	12	78	0.50	0.77	0.39	0.52
A*02:01	35	55	0.77	0.73	0.46	0.56
A*02:07	7	83	0.90	0.76	0.48	0.56
A*01:01	19	71	0.62	0.77	0.50	0.56
C*07:02	30	60	0.77	0.77	0.52	0.56
C*03:04	12	78	0.85	0.75	0.57	0.56
C*08:02	6	84	0.78	0.75	0.58	0.56
B*15:01	12	78	0.77	0.76	0.59	0.56
B*18:01	6	84	0.73	0.77	0.67	0.56

Datwani S, Kalikawe R, Mwimanzi K, Speckmaier S, Liang R, Sang Y, Waterworth R, Yaseen F, Lapointe HR, Barad E, DeMarco ML, Holmes DT, Simons J, Montaner JSG, Romney MG, Brumme ZL, Brockman MA. Dynamics of T-cell Responses Following COVID-19 mRNA Vaccination and Breakthrough Infection in Older Adults. *Pathogens and Immunity.* 2023;8(1):117–135. doi: 10.20411/pai.v8i1.613

Allele	N with[a]	N without	median with[b]	median without	*P*-value[c]	q-value
C*08:01	6	84	0.79	0.77	0.72	0.56
A*33:03	9	81	0.76	0.77	0.78	0.57
B*44:02	9	81	0.85	0.76	0.80	0.57
C*05:01	10	80	0.75	0.77	0.82	0.57
C*04:01	14	76	0.77	0.77	0.82	0.57
C*06:02	9	81	0.59	0.77	0.87	0.57
C*03:03	6	84	0.77	0.76	0.91	0.57
A*02:06	7	83	0.87	0.76	0.91	0.57

Results are sorted by statistical significance, with bold text denoting associations that meet the significance threshold after correction for multiple comparisons.
[a] N with/without: the N of participants expressing or not expressing the allele, respectively. Analysis was restricted to alleles expressed by a minimum of 5 participants.
[b] median with/without: the median spike-specific CD8+ T-cell frequencies after the third vaccine dose, in participants expressing or not expressing the allele, respectively.
[c] p-values are calculated using the Mann-Whitney U test.

Datwani S, Kalikawe R, Mwimanzi K, Speckmaier S, Liang R, Sang Y, Waterworth R, Yaseen F, Lapointe HR, Barad E, DeMarco ML, Holmes DT, Simons J, Montaner JSG, Romney MG, Brumme ZL, Brockman MA. Dynamics of T-cell Responses Following COVID-19 mRNA Vaccination and Breakthrough Infection in Older Adults. *Pathogens and Immunity.* 2023;8(1):117–135. doi: 10.20411/pai.v8i1.613

PATHOGENS AND IMMUNITY
IN ASSOCIATION WITH CASE WESTERN RESERVE UNIVERSITY AND THE RICHARD J. FASENMYER FOUNDATION

Supplementary Table 4. HLA A*02:01 and A*02:03 Binding Epitopes in SARS-CoV-2 Spike (Predicted Using NetMHCpan v4.1)

HLA	core	icore	EL_score	EL_rank	Cumulative rank	Strong Binder (SB)
HLA-A02:01	YLQPRTFLL	YLQPRTFLL	0.9712	0.0129	1	SB
HLA-A02:01	VLNDILSRL	VLNDILSRL	0.9385	0.028	2	SB
HLA-A02:01	TLDSKTQSL	TLDSKTQSL	0.915	0.0414	3	SB
HLA-A02:01	RLQSLQTYV	RLQSLQTYV	0.8738	0.0622	4	SB
HLA-A02:01	KIADYNYKL	KIADYNYKL	0.8646	0.0671	5	SB
HLA-A02:01	RLDKVEAEV	RLDKVEAEV	0.825	0.0899	6	SB
HLA-A02:01	LLFNKVTLA	LLFNKVTLA	0.8035	0.1053	7	SB
HLA-A02:01	HLMSFPQSA	HLMSFPQSA	0.7985	0.1085	8	SB
HLA-A02:01	VVFLHVTYV	VVFLHVTYV	0.7417	0.1528	9	SB
HLA-A02:01	ALNTLVKQL	ALNTLVKQL	0.6574	0.2258	10	SB
HLA-A02:01	RLNEVAKNL	RLNEVAKNL	0.6527	0.2303	11	SB
HLA-A02:01	FIAGLIAIV	FIAGLIAIV	0.6414	0.2409	12	SB
HLA-A02:01	GLTVLPPLL	GLTVLPPLL	0.6222	0.2593	13	SB
HLA-A02:01	NLNESLIDL	NLNESLIDL	0.6189	0.2624	14	SB
HLA-A02:01	SIIAYTMSL	SIIAYTMSL	0.58	0.2998	15	SB
HLA-A02:01	KLPDDFTGV	KLPDDFTGCV	0.5628	0.3198	16	SB
HLA-A02:01	KLNDLFTNV	KLNDLCFTNV	0.5334	0.3539	17	SB
HLA-A02:01	FLLHAPATV	FELLHAPATV	0.5132	0.3775	18	SB
HLA-A02:01	VLYENQKLI	VLYENQKLI	0.4959	0.3975	19	SB
HLA-A02:01	YLMSFPQSA	YHLMSFPQSA	0.4392	0.4737	20	SB
HLA-A02:01	SLNDILSRL	SVLNDILSRL	0.4151	0.5079	21	
HLA-A02:01	KLQDVVNQA	KLQDVVNQNA	0.4133	0.511	22	

PATHOGENS AND IMMUNITY
IN ASSOCIATION WITH CASE WESTERN RESERVE UNIVERSITY AND THE RICHARD J. FASENMYER FOUNDATION

HLA	core	icore	EL_score	EL_rank	Cumulative rank	Strong Binder (SB)
HLA-A02:01	RLITGLQSL	RLITGRLQSL	0.41	0.5166	23	
HLA-A02:01	TVYDPLPEL	TVYDPLQPEL	0.4092	0.5181	24	
HLA-A02:01	SLID-LQEL	SLIDLQEL	0.3954	0.5417	25	
HLA-A02:01	FTISVTTEI	FTISVTTEI	0.3759	0.575	26	
HLA-A02:01	VLSFELLHA	VLSFELLHA	0.3507	0.627	28	
HLA-A02:01	ELLHAPATV	ELLHAPATV	0.3364	0.6636	29	
HLA-A02:01	KLPDDFTGC	KLPDDFTGC	0.3349	0.6675	30	
HLA-A02:01	TLDSKTQSL	TLDSKTQSLL	0.2705	0.8537	31	
HLA-A02:01	TLDSKTQSL	TTLDSKTQSL	0.252	0.9172	32	
HLA-A02:01	SLSSTASAL	SLSSTASAL	0.2434	0.9509	33	
HLA-A02:01	KLNESLIDL	KNLNESLIDL	0.2428	0.9529	34	
HLA-A02:01	SLQTVTQQL	SLQTYVTQQL	0.2318	0.9958	35	
HLA-A02:01	NTQEVFAQV	NTQEVFAQV	0.2274	1.0149	36	
HLA-A02:01	VTWFHAIHV	VTWFHAIHV	0.2214	1.0425	37	
HLA-A02:01	SVTTEILPV	SVTTEILPV	0.2167	1.0636	38	
HLA-A02:01	FCNDPFLGV	FCNDPFLGV	0.2133	1.0794	39	
HLA-A02:01	FVSNGTWFV	FVSNGTHWFV	0.212	1.0853	40	
HLA-A02:01	GLQSLQTYV	GRLQSLQTYV	0.2107	1.091	41	
HLA-A02:01	VYDPLQPEL	VYDPLQPEL	0.1966	1.1573	42	
HLA-A02:01	YQPYRVVVL	YQPYRVVVL	0.189	1.1933	43	
HLA-A02:01	YLQPRTF-L	YLQPRTFL	0.1884	1.1964	44	
HLA-A02:01	FL-PFFSNV	FLPFFSNV	0.1844	1.2166	45	
HLA-A02:01	LLFNKV-TL	LLFNKVTL	0.1765	1.257	46	

HLA	core	icore	EL_score	EL_rank	Cumulative rank	Strong Binder (SB)

Datwani S, Kalikawe R, Mwimanzi K, Speckmaier S, Liang R, Sang Y, Waterworth R, Yaseen F, Lapointe HR, Barad E, DeMarco ML, Holmes DT, Simons J, Montaner JSG, Romney MG, Brumme ZL, Brockman MA. Dynamics of T-cell Responses Following COVID-19 mRNA Vaccination and Breakthrough Infection in Older Adults. *Pathogens and Immunity*. 2023;8(1):117–135. doi: 10.20411/pai.v8i1.613

HLA-A02:01	ALGKLQDVV	ALGKLQDVV	0.1733	1.2734	49	
HLA-A02:01	VLYE-NQKL	VLYENQKL	0.1725	1.2778	50	
HLA-A02:01	KQIYKTPPI	KQIYKTPPI	0.1717	1.2818	51	
HLA-A02:01	RVYSTGSNV	RVYSTGSNV	0.1643	1.3286	52	
HLA-A02:01	KIYSKHTPI	KIYSKHTPI	0.1618	1.3468	53	
HLA-A02:01	VLYQGVNCT	VLYQGVNCT	0.1603	1.3579	54	
HLA-A02:01	LQIPFAMQM	LQIPFAMQM	0.1573	1.3801	55	
HLA-A02:01	ELDSFKEEL	ELDSFKEEL	0.149	1.434	56	
HLA-A02:01	ALIPFAMQM	ALQIPFAMQM	0.146	1.4515	57	
HLA-A02:01	FLV-LLPLV	FLVLLPLV	0.1408	1.4827	58	
HLA-A02:01	GLQPRTFLL	GYLQPRTFLL	0.139	1.4934	59	
HLA-A02:01	GINASVVNI	GINASVVNI	0.1372	1.5054	60	
HLA-A02:01	FIEDLLFKV	FIEDLLFNKV	0.1371	1.5062	61	
HLA-A02:01	AQLTPTWRV	ADQLTPTWRV	0.1315	1.5466	62	
HLA-A02:01	ILDITPCSF	ILDITPCSF	0.1313	1.5481	63	
HLA-A02:01	YTNSFTRGV	YTNSFTRGV	0.1216	1.6254	64	
HLA-A02:01	VLYENQLIA	VLYENQKLIA	0.1203	1.6393	65	
HLA-A02:01	AIPNFTISV	AIPTNFTISV	0.1195	1.6483	66	
HLA-A02:01	VLHSQDLFL	VLHSTQDLFL	0.1166	1.6786	67	
HLA-A02:01	PLVDLPIGI	PLVDLPIGI	0.1158	1.6868	68	
HLA-A02:01	EQDKNTQEV	EQDKNTQEV	0.1128	1.721	69	
HLA-A02:01	ALLAGTITS	ALLAGTITS	0.1126	1.7229	70	
HLA-A02:01	YILGFIAGL	YIWLGFIAGL	0.1079	1.7765	71	

HLA	core	icore	EL_score	EL_rank	Cumulative rank	Strong Binder (SB)
HLA-A02:01	LITGRLQSL	LITGRLQSL	0.0982	1.9126	73	
HLA-A02:01	LLALHRSYL	LLALHRSYL	0.0959	1.942	74	

PATHOGENS AND IMMUNITY
IN ASSOCIATION WITH CASE WESTERN RESERVE UNIVERSITY AND THE RICHARD J. FASENMYER FOUNDATION

HLA	core	icore	EL_score	EL_rank	Cumulative rank	Strong Binder (SB)
HLA-A02:01	GIADYNYKL	GKIADYNYKL	0.0947	1.9582	75	
HLA-A02:01	QLNRALTGI	QLNRALTGI	0.0936	1.9735	76	
HLA-A02:01	YVTQQLIRA	YVTQQLIRA	0.0917	1.9986	77	
HLA-A02:03	VLNDILSRL	VLNDILSRL	0.9732	0.0058	1	SB
HLA-A02:03	LLFNKVTLA	LLFNKVTLA	0.8901	0.0391	2	SB
HLA-A02:03	YLQPRTFLL	YLQPRTFLL	0.8867	0.0406	3	SB
HLA-A02:03	HLMSFPQSA	HLMSFPQSA	0.8455	0.0611	4	SB
HLA-A02:03	ALNTLVKQL	ALNTLVKQL	0.8428	0.0626	5	SB
HLA-A02:03	VVFLHVTYV	VVFLHVTYV	0.8038	0.084	6	SB
HLA-A02:03	RLNEVAKNL	RLNEVAKNL	0.7887	0.0907	7	SB
HLA-A02:03	RLQSLQTYV	RLQSLQTYV	0.7798	0.0939	8	SB
HLA-A02:03	FIAGLIAIV	FIAGLIAIV	0.7635	0.0997	9	SB
HLA-A02:03	TLDSKTQSL	TLDSKTQSL	0.7588	0.103	10	SB
HLA-A02:03	SLNDILSRL	SVLNDILSRL	0.7044	0.1387	11	SB
HLA-A02:03	KLNDLFTNV	KLNDLCFTNV	0.6752	0.156	12	SB
HLA-A02:03	VLYENQKLI	VLYENQKLI	0.649	0.1785	13	SB
HLA-A02:03	RLIGRLQSL	RLITGRLQSL	0.5762	0.2509	14	SB
HLA-A02:03	KIADYNYKL	KIADYNYKL	0.5631	0.2647	15	SB
HLA-A02:03	SIIAYTMSL	SIIAYTMSL	0.5582	0.27	16	SB
HLA-A02:03	KLPDDFTGV	KLPDDFTGCV	0.5561	0.2722	17	SB
HLA-A02:03	RVYSTGSNV	RVYSTGSNV	0.5005	0.3381	18	SB
HLA-A02:03	SLSSTASAL	SLSSTASAL	0.4618	0.3881	19	SB
HLA-A02:03	YLMSFPQSA	YHLMSFPQSA	0.4561	0.3955	20	SB
HLA-A02:03	KLQDVVQNA	KLQDVVNQNA	0.4524	0.4003	21	SB
HLA-A02:03	TVYDPLQEL	TVYDPLQPEL	0.4446	0.4133	22	SB

Datwani S, Kalikawe R, Mwimanzi K, Speckmaier S, Liang R, Sang Y, Waterworth R, Yaseen F, Lapointe HR, Barad E, DeMarco ML, Holmes DT, Simons J, Montaner JSG, Romney MG, Brumme ZL, Brockman MA. Dynamics of T-cell Responses Following COVID-19 mRNA Vaccination and Breakthrough Infection in Older Adults. *Pathogens and Immunity.* 2023;8(1):117–135. doi: 10.20411/pai.v8i1.613

PATHOGENS AND IMMUNITY
IN ASSOCIATION WITH CASE WESTERN RESERVE UNIVERSITY AND THE RICHARD J. FASENMYER FOUNDATION

HLA	core	icore	EL_score	EL_rank	Cumulative rank	Strong Binder (SB)
HLA-A02:03	KIYSKHTPI	KIYSKHTPI	0.4398	0.4213	23	SB
HLA-A02:03	FTISVTTEI	FTISVTTEI	0.4168	0.4596	24	SB
HLA-A02:03	FLLHAPATV	FELLHAPATV	0.4156	0.4617	25	SB
HLA-A02:03	NLNESLIDL	NLNESLIDL	0.4146	0.4634	26	SB
HLA-A02:03	LLALHRSYL	LLALHRSYL	0.4066	0.4767	27	SB
HLA-A02:03	RLDKVEAEV	RLDKVEAEV	0.4037	0.4814	28	SB
HLA-A02:03	VLSFELLHA	VLSFELLHA	0.399	0.4893	29	SB
HLA-A02:03	QLNRALTGI	QLNRALTGI	0.3819	0.5257	30	
HLA-A02:03	MIAQYTSAL	MIAQYTSAL	0.3605	0.577	31	
HLA-A02:03	YTNSFTRGV	YTNSFTRGV	0.346	0.6123	32	
HLA-A02:03	GINASVVNI	GINASVVNI	0.3306	0.6516	33	
HLA-A02:03	ELLHAPATV	ELLHAPATV	0.3173	0.6855	34	
HLA-A02:03	GLTVLPPLL	GLTVLPPLL	0.3164	0.6876	35	
HLA-A02:03	SLID-LQEL	SLIDLQEL	0.3022	0.7326	36	
HLA-A02:03	FL-PFFSNV	FLPFFSNV	0.2951	0.7573	37	
HLA-A02:03	KLPDDFTGC	KLPDDFTGC	0.2798	0.8117	38	
HLA-A02:03	LITGRLQSL	LITGRLQSL	0.2672	0.8597	39	
HLA-A02:03	SVTTEILPV	SVTTEILPV	0.2468	0.9406	40	
HLA-A02:03	SLQTVTQQL	SLQTYVTQQL	0.2465	0.9416	41	
HLA-A02:03	VLYQGVNCT	VLYQGVNCT	0.2414	0.9626	42	
HLA-A02:03	NTQEVFAQV	NTQEVFAQV	0.2378	0.9773	43	
HLA-A02:03	FVSNGTHFV	FVSNGTHWFV	0.2339	0.9934	44	
HLA-A02:03	KLINQFNSA	KLIANQFNSA	0.2302	1.0095	45	
HLA-A02:03	YQPYRVVVL	YQPYRVVVL	0.2263	1.0279	46	

Datwani S, Kalikawe R, Mwimanzi K, Speckmaier S, Liang R, Sang Y, Waterworth R, Yaseen F, Lapointe HR, Barad E, DeMarco ML, Holmes DT, Simons J, Montaner JSG, Romney MG, Brumme ZL, Brockman MA. Dynamics of T-cell Responses Following COVID-19 mRNA Vaccination and Breakthrough Infection in Older Adults. *Pathogens and Immunity.* 2023;8(1):117–135. doi: 10.20411/pai.v8i1.613

HLA-A02:03	FLHVTYVPA	FLHVTYVPA	0.2181	1.0662	47	
HLA-A02:03	AQKFNGLTV	AQKFNGLTV	0.2162	1.0747	48	
HLA-A02:03	GVFLHVTYV	GVVFLHVTYV	0.2084	1.1134	49	
HLA-A02:03	KQIYKTPPI	KQIYKTPPI	0.196	1.1821	50	
HLA-A02:03	FCNDPFLGV	FCNDPFLGV	0.1946	1.1896	51	
HLA-A02:03	VLYENQKLA	VLYENQKLIA	0.1931	1.1975	52	
HLA-A02:03	GLQSLQTYV	GRLQSLQTYV	0.1879	1.2252	53	
HLA-A02:03	LLFNKVT-L	LLFNKVTL	0.181	1.2617	54	
HLA-A02:03	LIVNNATNV	LIVNNATNV	0.1603	1.4034	55	
HLA-A02:03	VVIGIVNTV	VVIGIVNNTV	0.1552	1.4509	56	
HLA-A02:03	VLYEN-QKL	VLYENQKL	0.1541	1.4612	57	
HLA-A02:03	AIPNFTISV	AIPTNFTISV	0.1525	1.4752	58	
HLA-A02:03	IVNNATNVV	IVNNATNVV	0.1505	1.4939	59	
HLA-A02:03	AISSVLNDI	AISSVLNDI	0.1492	1.5057	60	
HLA-A02:03	YVTQQLIRA	YVTQQLIRA	0.1446	1.5469	61	
HLA-A02:03	QMAYRFNGI	QMAYRFNGI	0.1436	1.5557	62	
HLA-A02:03	LLINNATNV	LLIVNNATNV	0.1423	1.5671	63	
HLA-A02:03	TLDSKTQSL	TLDSKTQSLL	0.1415	1.5743	64	
HLA-A02:03	FQFCNDPFL	FQFCNDPFL	0.1385	1.6011	65	
HLA-A02:03	LIANQFNSA	LIANQFNSA	0.1382	1.6036	66	

HLA	core	icore	EL_score	EL_rank	Cumulative rank	Strong Binder (SB)
HLA-A02:03	ALGKLQDVV	ALGKLQDVV	0.138	1.6049	67	
HLA-A02:03	KLNESLIDL	KNLNESLIDL	0.1369	1.6147	68	
HLA-A02:03	KEIDRLNEV	KEIDRLNEV	0.1283	1.6881	69	
HLA-A02:03	AQFNGLTVL	AQKFNGLTVL	0.1209	1.7612	70	

Datwani S, Kalikawe R, Mwimanzi K, Speckmaier S, Liang R, Sang Y, Waterworth R, Yaseen F, Lapointe HR, Barad E, DeMarco ML, Holmes DT, Simons J, Montaner JSG, Romney MG, Brumme ZL, Brockman MA. Dynamics of T-cell Responses Following COVID-19 mRNA Vaccination and Breakthrough Infection in Older Adults. *Pathogens and Immunity.* 2023;8(1):117–135. doi: 10.20411/pai.v8i1.613

HLA-A02:03	RLPQGFSAL	RDLPQGFSAL	0.1209	1.7616	71
HLA-A02:03	SALGKLQDV	SALGKLQDV	0.1205	1.7656	72
HLA-A02:03	TLDSKTQSL	TTLDSKTQSL	0.1201	1.7703	73
HLA-A02:03	AQYTSALLA	AQYTSALLA	0.1198	1.7729	74
HLA-A02:03	IVFPNITNL	IVRFPNITNL	0.1196	1.7749	75
HLA-A02:03	AVRDPTLEI	AVRDPQTLEI	0.1188	1.7834	76
HLA-A02:03	VLNDILSRL	VLNDILSRL	0.1176	1.7953	77
HLA-A02:03	KIQDSLSST	KIQDSLSST	0.116	1.8168	78
HLA-A02:03	KIQDSLSTA	KIQDSLSSTA	0.1116	1.8837	79
HLA-A02:03	YLQPR-TFL	YLQPRTFL	0.1106	1.8977	80
HLA-A02:03	GISGINASV	GDISGINASV	0.11	1.9067	81
HLA-A02:03	QLSSNFGAI	QLSSNFGAI	0.1063	1.954	82
HLA-A02:03	ALIPFAMQM	ALQIPFAMQM	0.1041	1.9827	83
HLA-A02:03	LLH-APATV	LLHAPATV	0.1031	1.9958	84

Datwani S, Kalikawe R, Mwimanzi K, Speckmaier S, Liang R, Sang Y, Waterworth R, Yaseen F, Lapointe HR, Barad E, DeMarco ML, Holmes DT, Simons J, Montaner JSG, Romney MG, Brumme ZL, Brockman MA. Dynamics of T-cell Responses Following COVID-19 mRNA Vaccination and Breakthrough Infection in Older Adults. *Pathogens and Immunity.* 2023;8(1):117–135. doi: 10.20411/pai.v8i1.613

PATHOGENS AND IMMUNITY
IN ASSOCIATION WITH CASE WESTERN RESERVE UNIVERSITY AND THE RICHARD J. FASENMYER FOUNDATION

Supplementary Table 5. Strong HLA A*02:01 or A*02:03 Binding Epitopes in SARS-CoV-2 Spike (Predicted Using NetMHCpan v4.1)

Spike Epitope	A*02:01 (% Rank)	A*02:03 (% Rank)	Notes
VLNDILSRL	0.028	0.0058	
LLFNKVTLA	0.1053	0.0391	
YLQPRTFLL	0.0129	0.0406	
HLMSFPQSA	0.1085	0.0611	
ALNTLVKQL	0.2258	0.0626	
VVFLHVTYV	0.1528	0.084	
RLNEVAKNL	0.2303	0.0907	
RLQSLQTYV	0.0622	0.0939	
FIAGLIAIV	0.2409	0.0997	
TLDSKTQSL	0.0414	0.103	
SVLNDILSRL	1.3286	0.1387	Enhanced binding for A*02:03
KLNDLCFTNV	0.3539	0.156	
VLYENQKLI	0.3975	0.1785	
RLITGRLQSL	2	0.2509	Enhanced binding for A*02:03
KIADYNYKL	0.0671	0.2647	
SIIAYTMSL	0.2998	0.27	
KLPDDFTGCV	0.3198	0.2722	
RVYSTGSNV	1.942	0.3381	Enhanced binding for A*02:03
SLSSTASAL	0.9509	0.3881	Enhanced binding for A*02:03
YHLMSFPQSA	0.4737	0.3955	
KLQDVVNQNA	0.511	0.4003	Enhanced binding for A*02:03
TVYDPLQPEL	2	0.4133	Enhanced binding for A*02:03
KIYSKHTPI	1.3468	0.4213	Enhanced binding for A*02:03
FTISVTTEI	0.575	0.4596	Enhanced binding for A*02:03
FELLHAPATV	0.3775	0.4617	

Datwani S, Kalikawe R, Mwimanzi K, Speckmaier S, Liang R, Sang Y, Waterworth R, Yaseen F, Lapointe HR, Barad E, DeMarco ML, Holmes DT, Simons J, Montaner JSG, Romney MG, Brumme ZL, Brockman MA. Dynamics of T-cell Responses Following COVID-19 mRNA Vaccination and Breakthrough Infection in Older Adults. *Pathogens and Immunity.* 2023;8(1):117–135. doi: 10.20411/pai.v8i1.613

Spike Epitope	A*02:01 (% Rank)	A*02:03 (% Rank)	Notes
NLNESLIDL	0.2624	0.4634	
LLALHRSYL	1.942	0.4767	Enhanced binding for A*02:03
RLDKVEAEV	0.0899	0.4814	
VLSFELLHA	0.627	0.4893	Enhanced binding for A*02:03
GLTVLPPLL	0.2593	0.6876	Enhanced binding for A*02:01

Datwani S, Kalikawe R, Mwimanzi K, Speckmaier S, Liang R, Sang Y, Waterworth R, Yaseen F, Lapointe HR, Barad E, DeMarco ML, Holmes DT, Simons J, Montaner JSG, Romney MG, Brumme ZL, Brockman MA. Dynamics of T-cell Responses Following COVID-19 mRNA Vaccination and Breakthrough Infection in Older Adults. *Pathogens and Immunity.* 2023;8(1):117–135. doi: 10.20411/pai.v8i1.613
