# Supplementary material for: Dynamics of T-cell Responses Following COVID-19 mRNA Vaccination and Breakthrough Infection in Older Adults
Source: Pathog Immun. 2023 Nov 17;8(1):117–35. doi: 10.20411/pai.v8i1.613 (PMC10686373; doi:10.20411/pai.v8i1.613)
Supplement: Supplementary Figures 1–4 [file pai-8-117-s02.pdf]

PATHOGENS AND IMMUNITY
IN ASSOCIATION WITH CASE WESTERN RESERVE UNIVERSITY AND THE RICHARD J. FASENMYER FOUNDATION

## SUPPLEMENTARY FIGURES

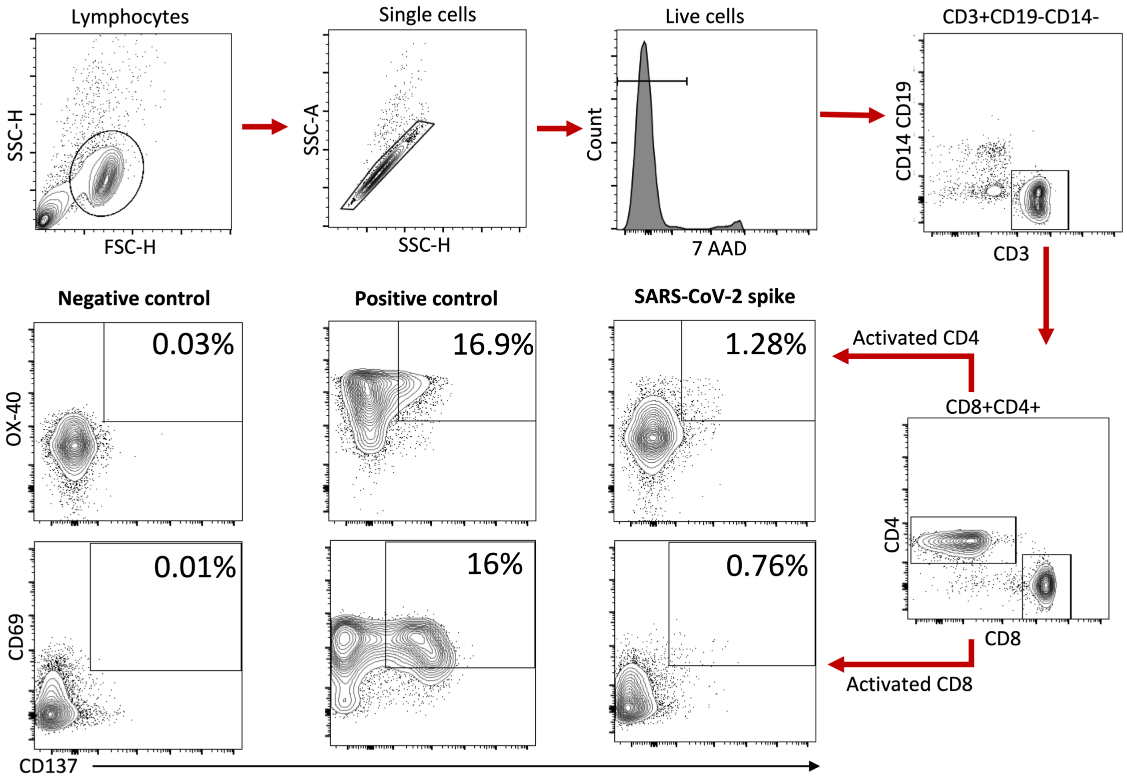


**Supplementary Figure 1**. **Gating strategy for quantification of SARS-CoV-2 Spike-specific CD4+ and CD8+ T-cells using an activation-induced marker assay.**

PATHOGENS AND IMMUNITY
IN ASSOCIATION WITH CASE WESTERN RESERVE UNIVERSITY AND THE RICHARD J. FASENMYER FOUNDATION

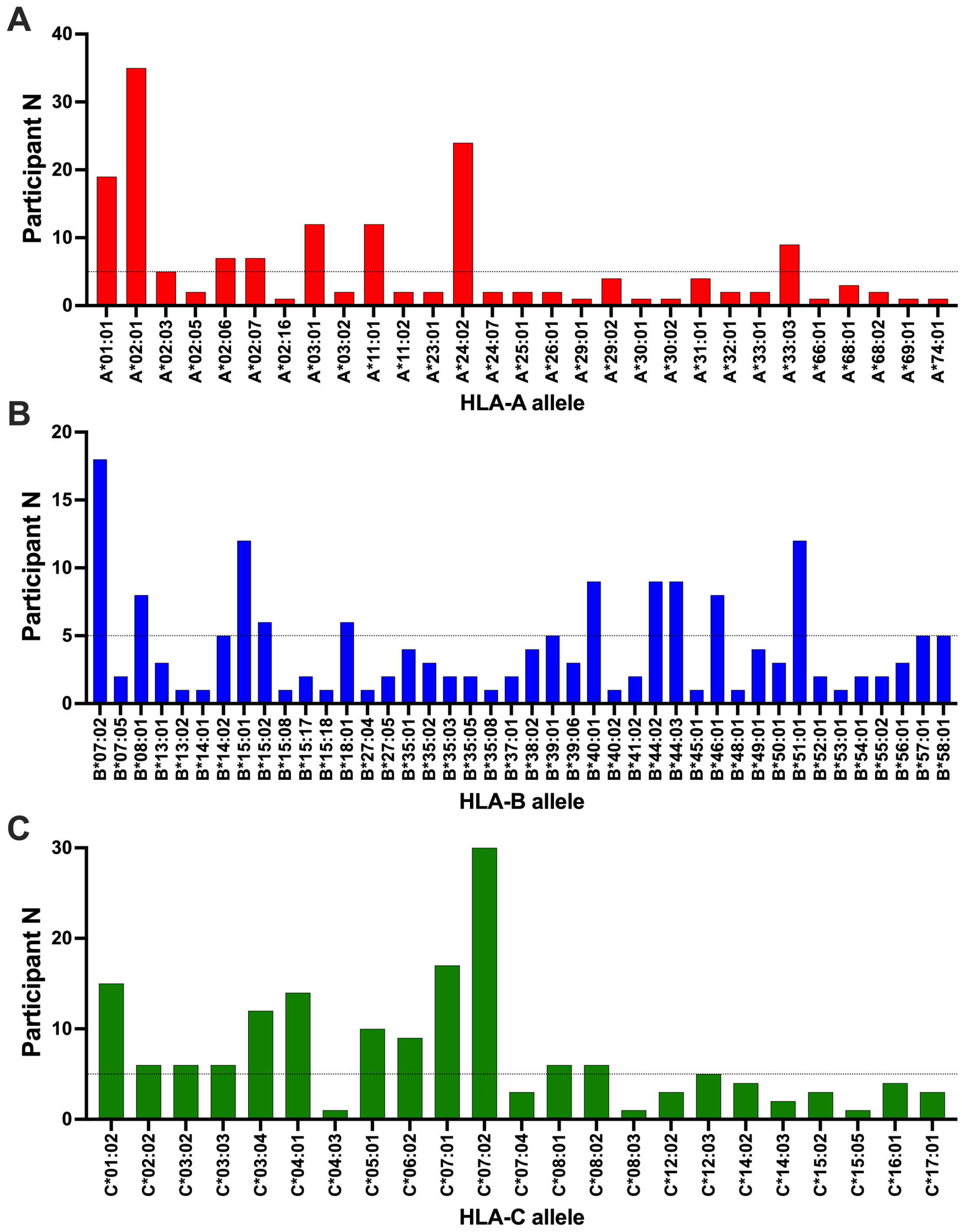


**Supplementary Figure 2. HLA-A, HLA-B, and HLA-C allele frequencies, reported as the number of participants expressing each allele in the overall cohort.** Alleles observed a minimum of 5 times, shown by the dotted line, were assessed for their relationship with SARS-CoV-2 Spike-specific CD8+ T-cell frequencies post-vaccination.

Datwani S, Kalikawe R, Mwimanzi K, Speckmaier S, Liang R, Sang Y, Waterworth R, Yaseen F, Lapointe HR, Barad E, DeMarco ML, Holmes DT, Simons J, Montaner JSG, Romney MG, Brumme ZL, Brockman MA. Dynamics of T-cell Responses Following COVID-19 mRNA Vaccination and Breakthrough Infection in Older Adults. *Pathogens and Immunity.* 2023;8(1):117–135. doi: 10.20411/pai.v8i1.613

PATHOGENS AND IMMUNITY
IN ASSOCIATION WITH CASE WESTERN RESERVE UNIVERSITY AND THE RICHARD J. FASENMYER FOUNDATION

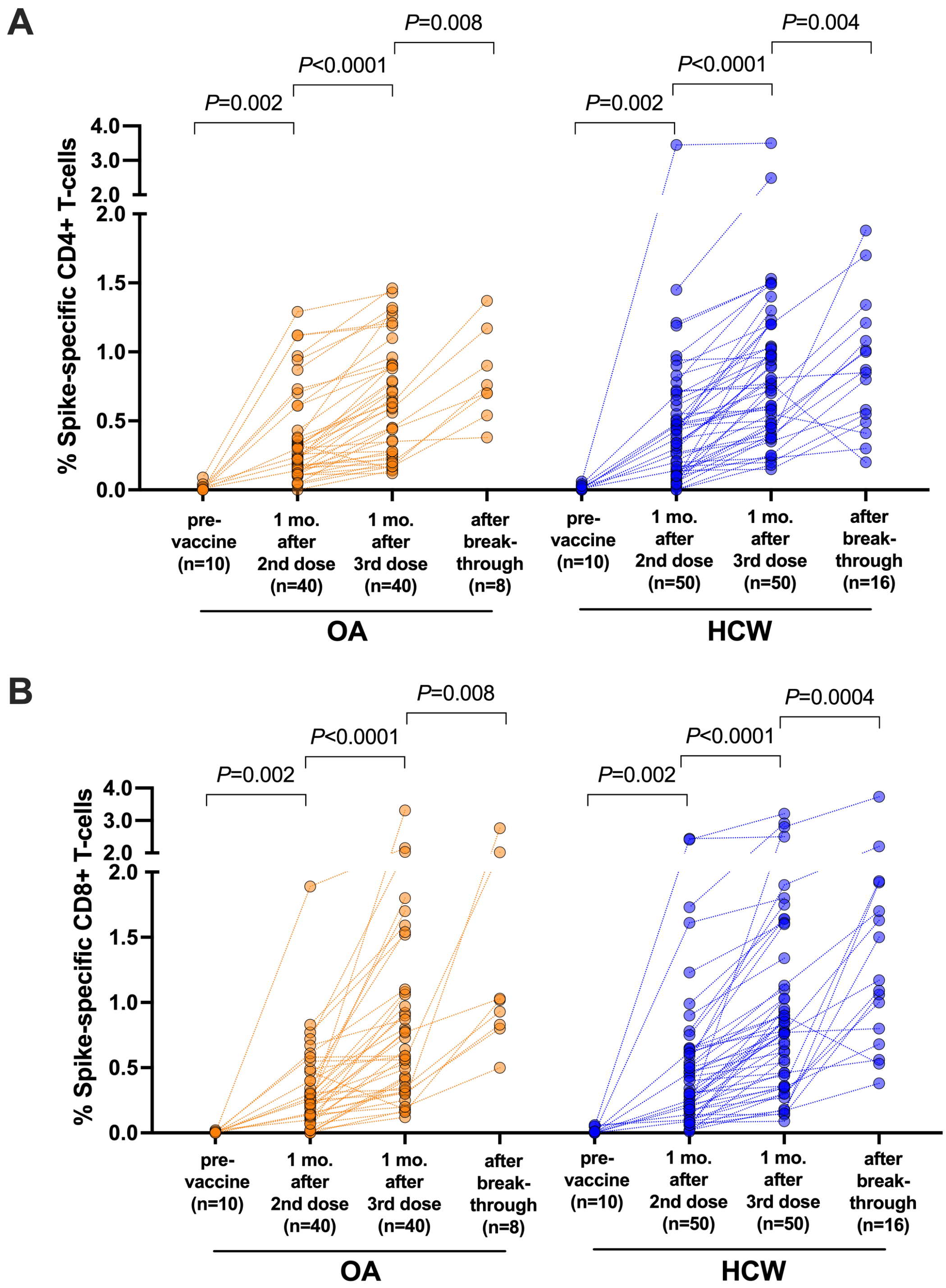


**Supplementary Figure 3. Longitudinal view of SARS-CoV-2 Spike-specific T-cell frequencies before and after vaccination and subsequent breakthrough infection.** These are the same data as shown in Figure 1 but plotted longitudinally by participant. *Panel A:* CD4+ T-cell frequencies. *Panel B:* CD8+ T-cell frequencies. Older Adults (OA) are in orange; younger Health Care Workers (HCW) are in blue. *P*-values are calculated using the Wilcoxon matched pairs test.

Datwani S, Kalikawe R, Mwimanzi K, Speckmaier S, Liang R, Sang Y, Waterworth R, Yaseen F, Lapointe HR, Barad E, DeMarco ML, Holmes DT, Simons J, Montaner JSG, Romney MG, Brumme ZL, Brockman MA. Dynamics of T-cell Responses Following COVID-19 mRNA Vaccination and Breakthrough Infection in Older Adults. *Pathogens and Immunity.* 2023;8(1):117–135. doi: 10.20411/pai.v8i1.613

PATHOGENS AND IMMUNITY
IN ASSOCIATION WITH CASE WESTERN RESERVE UNIVERSITY AND THE RICHARD J. FASENMYER FOUNDATION

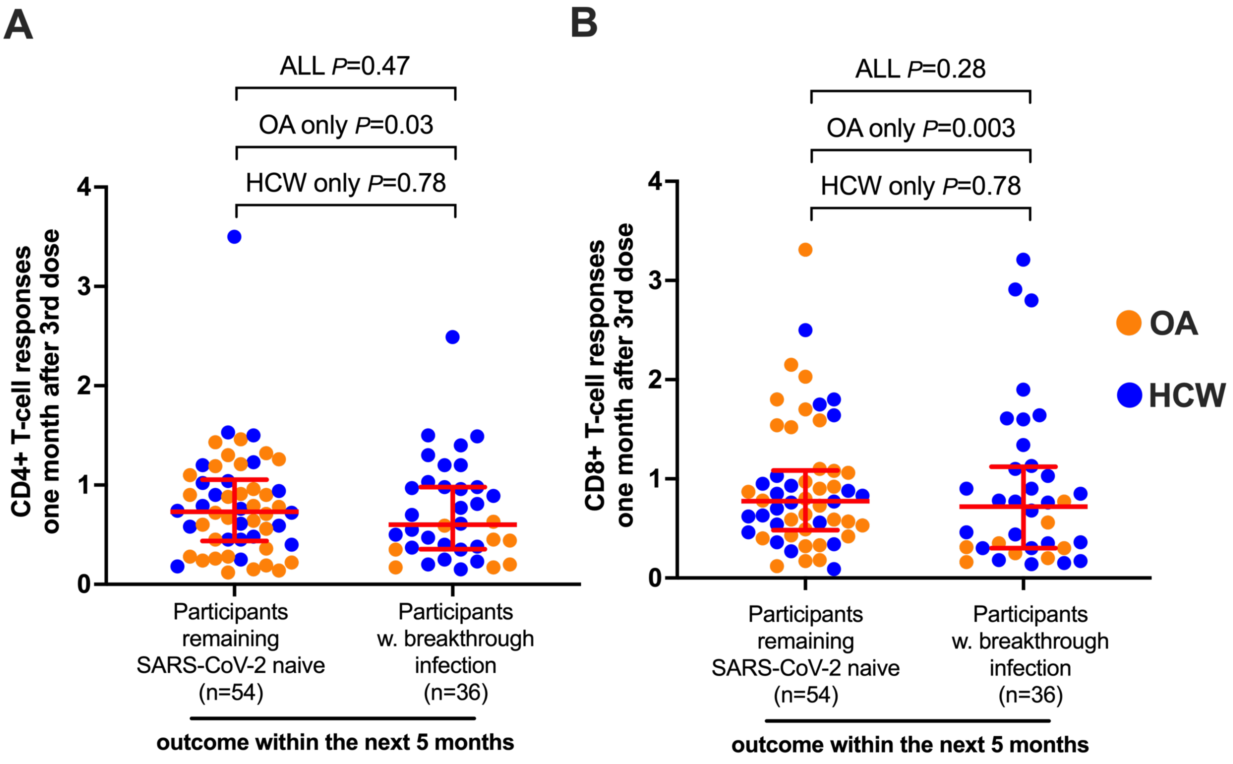


**Supplementary Figure 4. SARS-CoV-2 Spike-specific T-cell frequencies prior to breakthrough infection.** T-cell frequencies induced by 3 vaccine doses are shown for study participants who remained uninfected (n=54) or who became infected (n=36) within 5 months post-vaccination. *Panel A:* CD4+ T-cell frequencies. *Panel B:* CD8+ T-cell frequencies. Older Adults (OA) are in orange; younger Health Care Workers (HCW) are in blue. *P*-values are calculated using the Wilcoxon matched pairs test.

Datwani S, Kalikawe R, Mwimanzi K, Speckmaier S, Liang R, Sang Y, Waterworth R, Yaseen F, Lapointe HR, Barad E, DeMarco ML, Holmes DT, Simons J, Montaner JSG, Romney MG, Brumme ZL, Brockman MA. Dynamics of T-cell Responses Following COVID-19 mRNA Vaccination and Breakthrough Infection in Older Adults. *Pathogens and Immunity.* 2023;8(1):117–135. doi: 10.20411/pai.v8i1.613
